# Cocaine and amphetamine regulated transcript (CART) mediates sex differences in binge drinking through central taste circuits

**DOI:** 10.1038/s41386-023-01712-2

**Published:** 2023-08-22

**Authors:** Xavier J. Maddern, Bethany Letherby, Sarah S. Ch’ng, Amy Pearl, Andrea Gogos, Andrew J. Lawrence, Leigh C. Walker

**Affiliations:** 1 Florey Institute of Neuroscience and Mental Health, Parkville, VIC 3052 Australia; 2 Florey Department of Neuroscience and Mental Health, University of Melbourne, Parkville, VIC 3052 Australia

**Keywords:** Reward, Preclinical research

## Abstract

The neuropeptide cocaine- and amphetamine-regulated transcript (CART) has been implicated in alcohol consumption and reward behaviours, yet mechanisms mediating these effects have yet to be identified. Using a transgenic CART knockout (KO) mouse line we uncovered a sexually dimorphic effect of CART in binge drinking, with male CART KO mice increasing intake, whilst female CART KO mice decreased their alcohol intake compared to controls. Female CART KO mice show greater sensitivity to bitter solutions that can be overshadowed through addition of a sweetener, implicating taste as a factor. Further we identify that this is not driven through peripherally circulating sex hormones, but the central nucleus of the amygdala (CeA) is a locus where CART contributes to the regulation of alcohol consumption, with CeA CART neutralisation specifically reducing plain alcohol, but not sweetened alcohol consumption in female mice. These findings may have implications for the development of sex-specific treatment options for alcohol use disorders through targeting the CART system.

## Introduction

Alcohol use contributes to ~3 million global deaths annually, with alcohol misuse accounting for 5.1% of the global disease burden [1]. Binge drinking is the most common form of alcohol misuse in adolescents and young adults [2], and is often promoted by stress, social influences, pleasure/reward and taste [3–6]. Concerningly, binge drinking is a strong predictor and risk factor of the future development of alcohol dependence [7, 8].

Numerous sex differences have been reported in alcohol misuse and dependence. Historically, men exhibit elevated rates of alcohol use, binge drinking and alcohol use disorder (AUD) compared to women [9–13]. However, these rates have converged significantly over the past two decades, predominantly due to an increase in problematic alcohol use behaviours in women [9, 11–14]. This is especially noteworthy as women are more vulnerable to alcohol-induced health consequences, including physical, cognitive and mental health [9, 15, 16]. Importantly, greater attention has recently been directed toward studying sex differences in maladaptive alcohol use behaviours. Interestingly, several neuromodulatory systems differentially regulate alcohol consumption depending upon sex, including β-endorphin [17], orexin [18], and signalling via the κ-opioid receptor [19].

The neuropeptide cocaine- and amphetamine-regulated transcript (CART) is encoded by the *Cartpt* gene [20, 21]. There are two biologically active forms of CART in humans, CART_42-89_ and CART_49-89_, which correspond to CART_55-102_ and CART_62-102_ in rodents [22]. CART has been implicated in a diverse range of physiological and pathophysiological functions, including energy balance, depression, anxiety, and reward related behaviours, including AUD, for review see [23], which is reflected in its expression throughout the brain, including high abundance in metabolism, stress and reward circuits [24–30]. However, due to limitations in manipulating the CART system, it has remained relatively understudied. In humans, polymorphisms of the *Cartpt* gene are associated with AUD in a Korean male population [31], while in preclinical models, CART has been associated in alcohol consumption and seeking [30, 32–36]. CART mediates a number of factors that promote alcohol consumption, including stress/anxiety, reward seeking/motivation, taste and social interaction [22, 33, 34, 37–45]; however, the behavioural links of how CART may drive binge alcohol consumption have yet to be determined.

Given the interplay between CART and sex steroid hormones [46, 47], the rising incidence of risky alcohol drinking in women [48], and the known roles of CART driving factors that promote binge drinking [22, 33, 34, 37–45], we investigated potential sex differences in the role of CART in binge drinking, and the potential underlying behavioural factors driving excessive alcohol consumption. Our findings demonstrate a sexually dimorphic effect of CART in binge drinking and highlight a novel, sex-specific behavioural mechanism in females, whereby CART mediates binge drinking through changes in bitter taste sensitivity, in part through CART signalling in the CeA.

## Methods and materials

### Animals

CART KO and WT littermates were bred on a C57BL6J background at the Florey Institute of Neuroscience and Mental Health, with mutants identified using polymerase chain reaction and immunohistochemistry procedures (see Supplementary Materials and Methods; Fig. S1). Parental stock was kindly donated by Professor Herbert Herzog (Garvan Institute, Sydney) [49–51] and C57BL6J mice acquired from the Australian Resource Centre (ARC, Perth, Australia). All experiments were performed in accordance with the Prevention of Cruelty to Animals Act (2004), under the guidelines of the National Health and Medical Resource Council (NHMRC) Australian Code of Practice for the Care and Use of Animals for Experimental Purposes (2013) and approved by the Florey Institute of Neuroscience and Mental Health Animal Ethics Committee.

### Experiment 1: Binge drinking

Mice were trained to consume high levels of either 10% v/v ethanol (*n* = 8 WT males, *n* = 10 CART KO males, *n* = 9 WT females, *n* = 9 CART KO females), 5% w/v sucrose (*n* = 8 WT males, *n* = 8 CART KO males, *n* = 9 WT females, *n* = 8 CART KO females), or 10% v/v ethanol +5% w/v sucrose (*n* = 5 WT males, *n* = 5 CART KO males, *n* = 6 WT females, *n* = 5 CART KO females) using a binge drinking procedure [52, 53]. Here, mice had home-cage access to ethanol and/or sucrose 3 times/week, 3 h into the dark phase for 2 h. Once drinking levels were stable (~10 sessions), mice were tested in an extended 4 h session, with intake converted to grams per kilogram (g/kg).

### Experiment 2: Continuous alcohol intake and preference

CART KO (*n* = 8 males, *n* = 8 females) and WT (*n* = 9 males, *n* = 10 females) mice had continuous access to a bottle containing ethanol and a bottle containing water (two-bottle choice) for 10 weeks, with increasing concentrations of ethanol (v/v; 5% 3 weeks, 10% 3 weeks, 15% 2 weeks, and 20% 2 weeks) and intake measured daily at 10:00 am [54]. To determine whether the taste of ethanol contributed to altered consumption in female mice, bottles of ethanol were then supplemented with sucrose (1–5% w/v) and intake was measured daily. Following this, mice underwent testing for anxiety-like (light-dark box) and anhedonia-like (saccharin preference test; SPT, Porsolt swim test; PST) behaviour and were compared to age and genotype matched controls with water access only (see Supplementary Materials and Methods).

### Experiment 3: Taste preferences

Separate cohorts of male (*n* = 9 CART KO, *n* = 9 WT) and female mice (*n* = 8 CART KO, *n* = 10 WT) were tested for consumption and preference of a range of tastants; sweet (saccharin; 0.001–0.1% w/v), salt (NaCl; 35–600 mM), sour (citric acid; 1–30 mM) and bitter (quinine; 0.001–0.1% w/v), increasing in concentration daily with a 3-day washout period between solutions. To determine whether a bitter taste profile contributes to the altered consumption in female CART KO mice, bottles of quinine (0.01% w/v) were subsequently supplemented with sucrose (1–5% w/v) and intake was measured daily.

### Experiment 4: Ovariectomy and binge drinking

To assess the potential influence of peripherally circulating sex steroid hormones on binge drinking, female CART KO mice underwent ovariectomy (*n* = 10) or SHAM (*n* = 10) surgery during binge drinking training (see Supplementary Materials and Methods for surgical details). After surgery, recovery and re-training, mice were tested in a 4-h extended alcohol session. Upon completion uteri were dissected and weighed to validate successful ovariectomy.

### Experiment 5: CeA CART signalling in binge drinking

Given the role of the CeA in taste perception [55–57] and dense expression of CART within the capsular and lateral divisions [30], we next sought to determine whether CeA CART signalling regulates alcohol binge drinking specifically in female mice. Male (*n* = 16) and female C57BL6J mice (*n* = 15) underwent bilateral CeA cannula implantation during binge drinking training (see Supplementary Materials and Methods). Following recovery, re-training and habituation, mice were tested under each treatment condition in a randomised counterbalanced manner. Directly prior to testing, mice were bilaterally infused with 0.5 µl/hemisphere of either CART antibody (rabbit anti-CART55-102; 1:500, #H-003-62, Phoenix Pharmaceuticals) or vehicle (normal rabbit serum, NRS, 1:500) at a rate of 0.25 µl/min into the CeA [30]. Mice were re-trained to baseline drinking levels before testing with the opposite treatment condition (~one week between test sessions).

To determine whether this effect in females was specific to the taste of alcohol, in a separate cohort of female mice we tested whether neutralisation of CeA CART signalling reduced 10% v/v ethanol consumption supplemented with 5% w/v sucrose. Female C57BL6J mice (*n* = 18) underwent the same experimental procedure detailed above, except were presented with a 10% v/v ethanol +5% w/v sucrose solution during training and test.

### Data analysis

Data were analysed by *students t*-test or Two-way ANOVA using Prism 9. See Supplementary Material for details (Tables S1–5).

## Results

### Experiment 1: Global CART KO has a sexually dimorphic effect on binge drinking

**Fig. 1:**
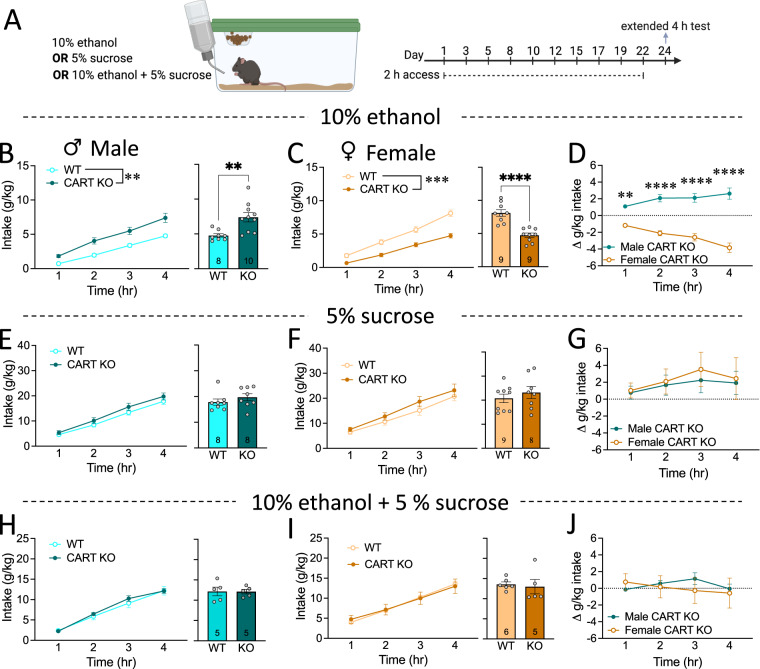
CART has a sexually dimorphic effect on binge drinking. **A** Schematic of experimental outline. **B** Male CART KO mice had increased cumulative (left) and total (right) while (**C**) female CART KO mice had decreased cumulative (left) and total (right) ethanol intake compared to WT littermates. **D** Δ intake of ethanol normalised to same-sex WT littermates revealed a sexually dimorphic role of CART in binge drinking. No differences in sucrose intake were seen in (**E**) male or (**F**) female CART KO mice compared to WT littermates, and (**G**) no sex difference in the role of CART in sucrose intake was observed. There were no differences in cumulative (left) and total (right) intake of ethanol supplemented with sucrose in (**H**) male or (**I**) female CART KO mice compared to WT littermates, and (**J**) no sex difference in the role of CART in sweetened ethanol intake was observed. Data presented as mean ± SEM. **p < 0.01, ***p < 0.001, ****p < 0.0001. *n* = 5–10/group. WT wildtype, KO knockout.

We first explored the effect of global CART KO on binge drinking in male and female mice (Fig. 1A, see Fig. S2 for training data). Male CART KO mice had increased cumulative 10% v/v ethanol intake during the binge drinking test (main effect of genotype, *p* = 0.0018; Fig. 1B) compared to WT littermates. Bonferroni *post-hoc* analysis revealed increased cumulative ethanol intake in male CART KO mice after 2 h, 3 h and 4 h (all *p*’s < 0.005; Fig. 1B). Male CART KO mice also had increased total ethanol intake (*p* = 0.004; Fig. 1B). Contrastingly, female CART KO mice had reduced cumulative ethanol intake (main effect of genotype, *p* = 0.0001; Fig. 1C) compared to their WT littermates. Bonferroni *post-hoc* analysis found cumulative ethanol intake in female CART KO mice to be reduced after 2 h, 3 h and 4 h (all *p*’s < 0.001; Fig. 1C). Female CART KO mice also had reduced total ethanol intake compared to WT littermates (*p* < 0.0001; Fig. 1C). Assessment of change in ethanol intake normalised to sex matched WT littermates (Δ intake) revealed a sexual dimorphism in binge drinking, with increased intake in male CART KO mice compared to female CART KO mice (*p* < 0.0001; Fig. 1D). Bonferroni *post-hoc* analysis revealed this difference persisted throughout the binge drinking test (all *p* < 0.001; Fig. 1D).

Male CART KO and WT littermates displayed no difference in cumulative 5% w/v sucrose intake (no main effect of genotype, *p* = 0.260; Fig. 1E) or total sucrose intake (*p* = 0.720; Fig. 1E). Similarly, female CART KO and WT mice had comparable cumulative (no main effect of genotype, *p* = 0.324; Fig. 1F) and total (*p* = 0.813; Fig. 1F) sucrose intake levels. There was no sex difference in Δ sucrose intake relative to sex matched WT littermates in CART KO mice (*p* = 0.702; Fig. 1G).

Male CART KO and WT littermates had similar levels of cumulative (no main effect of genotype, *p* = 0.644; Fig. 1H) and total (*p* = 0.963; Fig. 1H) intake of 10% v/v ethanol supplemented with 5% w/v sucrose. Interestingly, there was no difference in cumulative 10% v/v ethanol +5% w/v sucrose consumption between female CART KO and WT mice (no main effect of genotype, *p* = 0.987; Fig. 1I), or total intake (*p* = 0.749; Fig. 1I). Additionally, no sex difference was found in Δ intake relative to sex matched WT littermates (*p* = 0.807; Fig. 1J).

### Experiment 2: Global CART KO in female mice reduces ethanol consumption, which can be elevated through sucrose supplementation

**Fig. 2:**
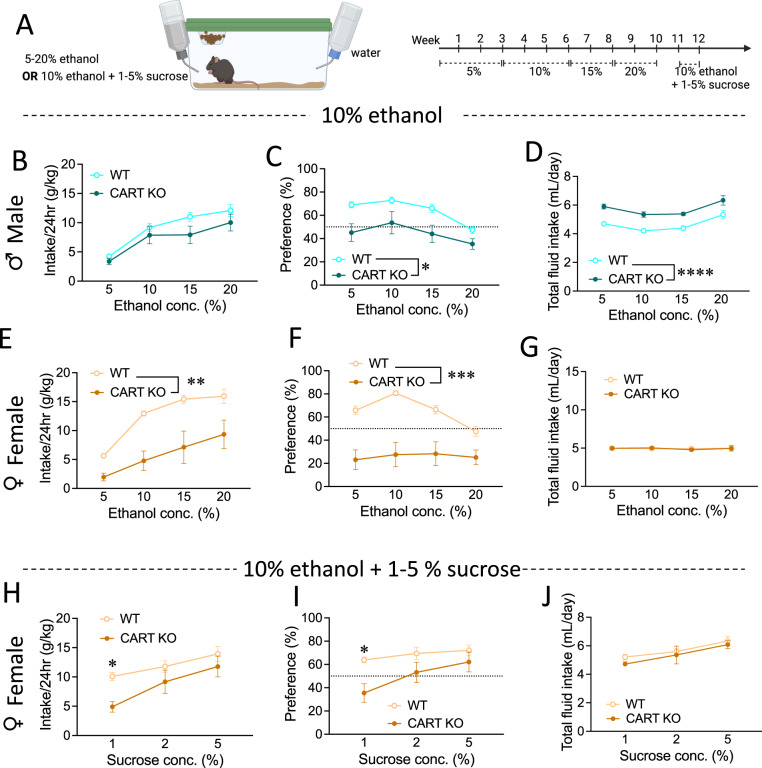
CART KO reduces ethanol consumption and preference in female mice that can be elevated via sucrose supplementation. **A** Schematic of experimental outline. Male CART KO and WT mice showed no difference in (**B**) ethanol intake, but CART KO mice had (**C**) reduced ethanol preference, (**D**) and increased total fluid intake compared to WT mice in a continuous access procedure. Female CART KO mice had reduced (**E**) ethanol intake and (**F**) preference, (**G**) no difference in total fluid intake compared to WT littermates a continuous access procedure. Female CART KO mice had reduced intake (**H**) and preference (**I**) for alcohol supplemented with sucrose, which was specific to 1% w/v sucrose supplementation, and (**J**) no differences in total fluid intake compared to WT mice. Data presented as mean ± SEM. *p < 0.05, **p < 0.01, ***p < 0.001, ****p < 0.0001 *n* = 8–10/group. WT wildtype, KO knockout.

We further probed whether similar changes in alcohol consumption were observed during continuous access (Fig. 2A). Male CART KO and WT mice had comparable levels of ethanol intake during the two-bottle choice procedure across concentrations (no main effect of genotype, *p* = 0.148; Fig. 2B), but a reduced preference toward ethanol was found in male CART KO mice (main effect of genotype, *p* = 0.012; Fig. 2C). This was driven by greater fluid intake in male CART KO mice (main effect of genotype, *p* < 0.0001; Fig. 2D). Bonferroni *post-hoc* analysis found total fluid intake in male CART KO mice to be increased for all ethanol concentrations (all *p*’s < 0.01; Fig. 2D).

In line with ethanol binge drinking, female CART KO mice had reduced ethanol intake compared to WT littermates in the continuous access paradigm (main effect of genotype, *p* = 0.0015; Fig. 2E). Bonferroni *post-hoc* analysis revealed female CART KO mice to have decreased ethanol intake at 10%, 15%, and 20% ethanol concentrations (all *p*’s < 0.05; Fig. 2E). Ethanol preference was also reduced in female CART KO mice (main effect of genotype, *p* = 0.0002; Fig. 2F) across all ethanol concentrations (all *p*’s < 0.05; Fig. 2F). No difference in total fluid intake between female CART KO and WT mice was observed (no main effect of genotype, *p* = 0.959; Fig. 2G).

Several factors can alter ethanol consumption including anxiety, depression and taste [5, 58–62]. To determine whether these may underpin the genotype differences observed in female mice, we tested for anxiety-like behaviour (L/D box) and anhedonia-like behaviour (SPT and PST). No differences were seen in anxiety- or anhedonia-like behaviour in naïve or alcohol experienced, female CART KO and WT mice, suggesting these do not underpin differences in alcohol consumption (Fig. S3).

Given no difference in binge drinking was observed with sweetened alcohol in experiment 1, we next assessed whether supplementing alcohol with sucrose would elevate the reduced intake and preference in female mice in a dose related manner. Female CART KO mice had reduced intake of sucrose supplemented ethanol compared to WT littermates (main effect of genotype, *p* = 0.0447; Fig. 2H). However, decreased ethanol intake was only present at 1% w/v sucrose supplementation (*p* = 0.014; Fig. 2H). Female CART KO mice also had a reduced preference toward sucrose supplemented ethanol compared to WT littermates (main effect of genotype, *p* = 0.0435; Fig. 2I), which was specific to 1% w/v sucrose supplementation (*p* = 0.0247; Fig. 2I). Female CART KO and WT mice also had comparable levels of total fluid intake across solutions (no main effect of genotype, *p* = 0.3047; Fig. 2J). Together these data suggest that increasing the palatability of ethanol can elevate consumption and preference in female CART KO mice.

### Experiment 3. Reductions in alcohol consumption in female CART KO mice are driven through sensitivity to bitter taste

**Fig. 3:**
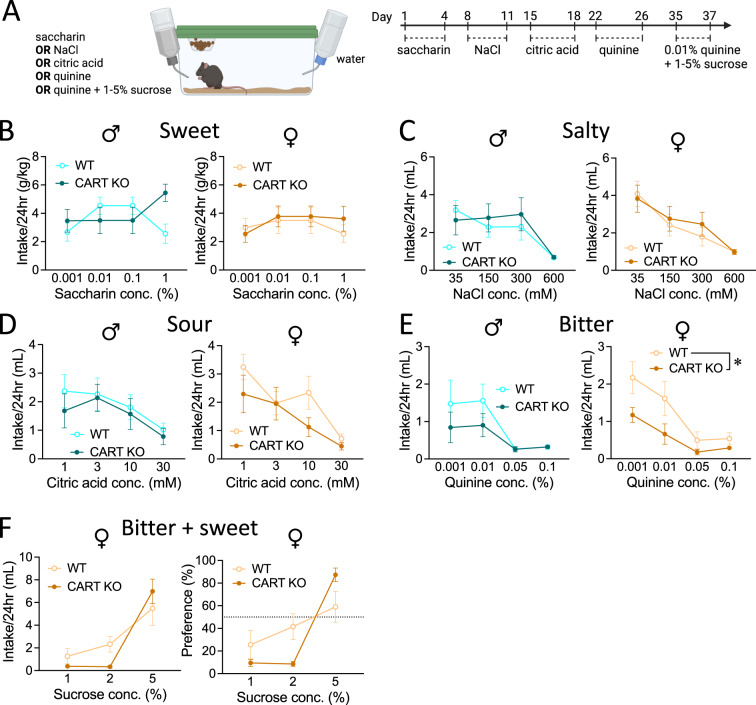
Female CART KO mice have reduced intake of quinine, that can be elevated through sucrose supplementation. **A** Experimental timeline. Male (left) and female (right) CART KO and WT mice have comparable intake of (**B**) sweet, (**C**) salty and (**D**) sour solutions. **E** Male CART KO and WT mice also have similar intake of quinine (left), whilst female CART KO mice have reduced intake of quinine compared to female WT mice (right). **F** Quinine intake (left) and preference (right) could be elevated to WT levels in CART KO female mice through sucrose supplementation. Data presented as mean ± SEM. *p < 0.05. *n* = 8–10/group. D day, conc. concentration, mM millimolar, WT wildtype, KO knockout.

The four most widely recognised primary tastes are sweet, sour, salty and bitter [63]. The taste of ethanol depends on the concentration used, at lower concentrations (3–16% v/v) ethanol elicits a predominantly bitter taste with a much lower intensity taste of sweetness [64, 65]. Thus, we next sought to assess and compare the intake and preference toward a variety of different tastes between male and female KO and WT mice (Fig. 3A). Male CART KO and WT and female CART KO and WT mice had comparable intake of sweet (saccharin, Fig. 3B), salty (NaCl, Fig. 3C) and sour (citric acid, Fig. 3D) solutions (all *p*’s < 0.05). However, while male CART KO and WT mice had similar intake of the bitter solution (quinine, Fig. 3E), female CART KO mice had reduced intake of quinine compared to WT littermates (main effect of genotype, *p* = 0.0179; Fig. 3E). Interestingly, supplementation of 0.01% w/v quinine with sucrose elevated female CART KO mice quinine intake in line with WT littermates (no main effect of genotype, *p* = 0.2953; Fig. 3F) and preference (no main effect of genotype, *p* = 0.5166; Fig. 3F). For all preference data see Fig. S4.

### Experiment 4: Peripherally circulating sex steroid hormones do not alter binge drinking in female CART KO mice

**Fig. 4:**
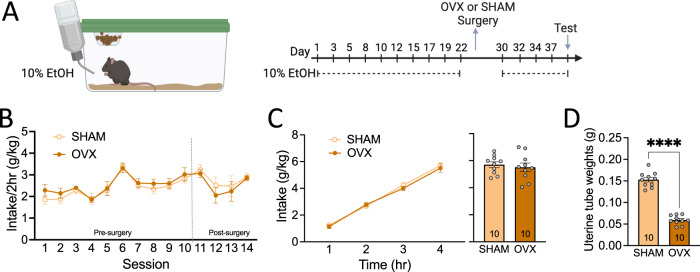
Peripherally circulating sex steroid hormones do not mediate binge drinking in female CART KO mice. **A** Timeline of experimental procedure with surgical intervention. **B** No differences in ethanol intake were observed during binge drinking training pre- and post-surgery between SHAM and OVX female CART KO mice. **C** OVX and SHAM surgery mice had comparable levels of cumulative (left) and total (right) intake during the binge drinking test. **D** OVX mice had reduced uterine tube weights compared to SHAM mice, validating surgery efficacy. Data presented as mean ± SEM. ****p < 0.0001. *n* = 10/group. OVX ovariectomised, KO knockout, hr hour.

Sex steroid hormones can influence alcohol intake in both sexes [66] and CART expression [46, 47]. Our results showed robust (>40%) reduction in alcohol intake in female CART KO mice across both binge and continuous access models. Although both paradigms showed robust results, we chose to pursue the binge model of alcohol intake as it leads to translationally relevant blood alcohol levels, rather than mice “grazing” on alcohol across a 24-h period. Consequently, we next examined whether peripherally circulating sex steroid hormones influence binge drinking in female CART KO mice through assessing the effect of ovariectomy on binge drinking (Fig. 4A). No differences in 10% v/v ethanol intake were observed pre- vs. post-surgery across SHAM and ovariectomized mice (*p’s* > 0.05; Fig. 4B), or during the extended 4 h test for cumulative (no main effect of surgery group, *p* = 0.6417; Fig. 4C) and total (*p* = 0.6161; Fig. 4C) ethanol intake. Ovariectomized mice had significantly reduced uterine tube weights compared to SHAM surgery mice (*p* < 0.0001; Fig. 4D), validating surgery efficacy.

### Experiment 5: Neutralisation of CeA CART signalling reduces binge drinking in female, but not male mice, which can be enhanced through sucrose supplementation

**Fig. 5:**
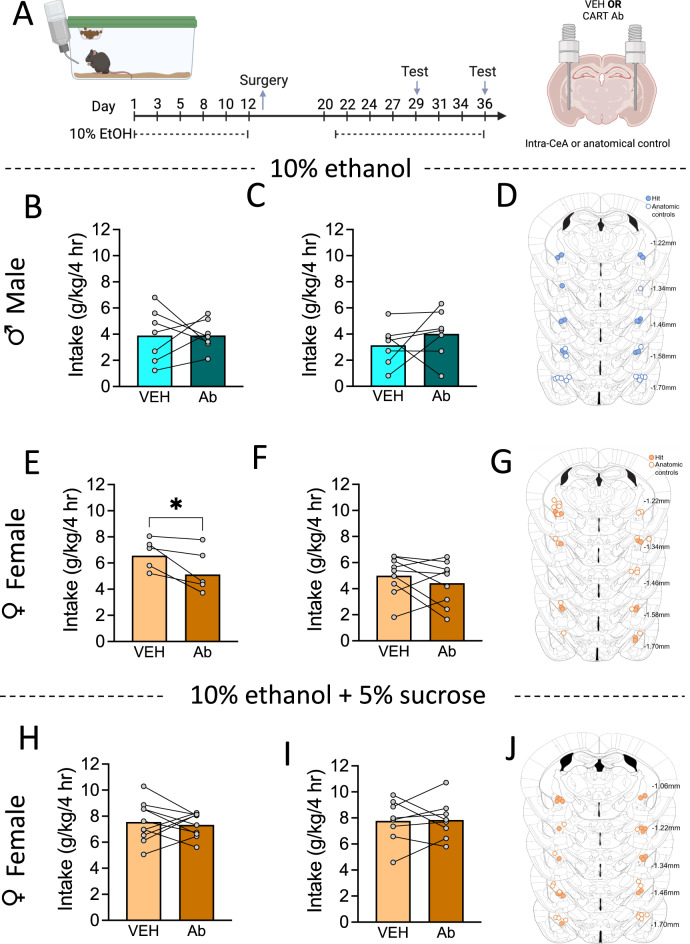
Neutralisation of CeA CART signalling reduces binge drinking of ethanol, but not sweetened ethanol in female mice. **A** Timeline of experimental procedure. No difference in binge drinking was seen in male mice following bilateral neutralisation of CART signalling in (**B**) the CeA or in (**C**) anatomic controls. **D** Cannula placements for male ethanol binge drinking, closed circles represent bilateral CeA administration, open circles represent anatomic controls. **E** Bilateral neutralisation of CART signalling in the CeA reduced binge drinking in female mice, (**F**) whilst no difference was observed in anatomic controls following CART Ab infusion. **G** Cannula placements for female ethanol binge drinking, closed circles represent bilateral CeA administration, open circles represent anatomic controls. No difference in binge drinking of sucrose supplemented ethanol were seen following neutralisation of CART signalling in (**H**) the CeA or in (**I**) anatomic controls. **J** Cannula placements for sweetened ethanol binge drinking in female mice, closed circles represent bilateral CeA administration, open circles represent anatomic controls. Data are presented as mean ± SEM. *p < 0.05. *n* = 6–10/group. Ab antibody, VEH vehicle, hr hours.

The CeA is critically involved in taste perception [55–57] and densely expresses CART in the capsular and lateral divisions [30], yet how CeA CART may influence alcohol intake through changes in taste remain unknown. To test whether CeA CART signalling regulates binge drinking we neutralised CART signalling in the CeA directly prior to a binge drinking test [30] (Fig. 5A). In male mice, intra-CeA CART neutralisation had no effect on total ethanol intake in the binge drinking test when cannula were placed either within (*p* = 0.9875; Fig. 5B) or adjacent (anatomic control; *p* = 0.3728; Fig. 5C) to the CeA. Figure 5D shows cannula placements. Interestingly, in female mice, intra-CeA CART neutralisation reduced total ethanol intake in the binge drinking test (*p* = 0.0183; Fig. 5E). Importantly there was no effect of CART neutralisation on binge drinking in mice with at least one cannula placed adjacent to the CeA (*p* = 0.3183; Fig. 5F). Figure 5G shows cannula placements. To determine whether this action was driven by the taste of alcohol in female mice, we supplemented 10% v/v ethanol with 5% w/v sucrose and repeated the experiment. Neutralisation of CeA CART signalling had no effect on binge drinking when cannula were placed either within (*p* = 0.6824; Fig. 5H) or adjacent (*p* = 0.9034; Fig. 5I) to the CeA. Figure 5J shows cannula placements.

## Discussion

Here we show a sexual dimorphism in the role of CART in alcohol binge drinking. Moreover, we identify a novel behavioural mechanism in which CART mediates alcohol intake through alterations in bitter taste sensitivity, specifically in female mice, driven in part through CART signalling in the CeA. Together, these data further highlight the importance of taste, especially bitter taste, in alcohol intake and preference, and that CART signalling, including within the CeA, alters sensitivity to bitter taste in a sex-specific manner to regulate alcohol intake in female mice.

This study expands upon the literature linking CART to numerous alcohol-related behaviours, including consumption and preference [32, 33], withdrawal [34] and alcohol seeking [30, 35, 36], highlighting a role in binge drinking. Interestingly, our results demonstrate a sexually dimorphic role for CART signalling in binge drinking of plain alcohol, but not sweetened alcohol, or sucrose alone. Specifically, male CART KO mice showed heightened alcohol consumption, while female CART KO mice showed reduced alcohol consumption in a modified drinking in the dark paradigm. In contrast, both sexes show reduced ethanol preference in a continuous access paradigm, with females also showing reduced intake, similar to previous reports [33]. However, in contrast to previous literature [33], CART KO male mice also showed elevated levels of total fluid intake. This may suggest that CART KO alters ethanol intake through sex-specific mechanisms; in male mice via a dysregulation in fluid intake when ethanol is available, whilst in female mice, due to an enhanced bitter taste sensitivity. This likely contributes to elevated alcohol consumption during the binge drinking procedure, where only alcohol is available. However, we did not robustly observe increased fluid consumption with other tastants (saccharin, citric acid or quinine) in a similar choice protocol. The discrepancy in findings to previous research may be attributable to the use of different CART KO mouse lines and procedures to assess ethanol intake [67].

Peripherally circulating sex hormones can influence alcohol-related behaviours [66, 68], and neuropeptide expression in the brain [69]. CART and estrogen have been shown to directly influence each other. For example, estradiol increases CART immunoreactivity in the hypothalamus [70], while CART-induced accumbal dopamine release is not observed in ovariectomised rats [71]. Interestingly, ovariectomy had no effect on alcohol intake in female CART KO mice, suggesting the sexual dimorphism in CART regulating binge drinking is not influenced by peripherally circulating sex hormones and that it may be centrally mediated. Indeed, there are known sex-related structural, morphological and neurochemical differences of the brain [72, 73] and known sex differences in the effects of alcohol on the brain directly [74–82]. Our data provide further evidence of sex differences in the neurobiological mechanisms underpinning excessive alcohol intake.

CART is critically involved in alcohol-induced anxiety-like behaviour, anhedonia-like behaviour and taste aversion [30, 34, 37], and these factors can all influence alcohol consumption. Therefore, we employed a battery of behavioural tests in naïve and alcohol experienced female mice to determine their contribution to observed changes in drinking behaviour. CART KO female mice displayed no difference in anxiety-like or anhedonia-like behaviour compared to WT littermates; however, we did observe differences in taste preference, specifically a heightened bitter taste sensitivity in female CART KO mice.

Taste is an important and often overlooked factor mediating alcohol preference, intake and use behaviours [5]. Taste is critical in driving intake and preference for both food and beverages, including alcohol [83–86]. For example, people with heightened perception of bitter taste intensity also have a stronger perceived intensity of alcohol and accordingly a lower intake [87–90]. Here we found female CART KO and WT mice had comparable levels of intake and preference for salty, sour and sweet solutions, but a specific reduction in consumption of a bitter solution (quinine) in CART KO female mice. Importantly, reduced alcohol and quinine intake could both be elevated in a dose-related manner through masking bitter taste with a sweetener. Further, no difference in binge drinking of sucrose, or alcohol supplemented with sucrose was observed. Previous research has reported no differences in sweet or bitter taste sensitivity between CART KO and WT mice in either sex [33], however, our study used a larger range and higher concentrations of different solutions. Further, central administration of exogenous CART peptide elicits a conditioned taste aversion [37], however, understanding of the central mechanisms driving CARTs role in taste aversion is limited.

Importantly, CART is expressed throughout several nodes of central taste circuitry, including the CeA [24, 25, 29]. Indeed, the CeA is a key brain region involved in taste perception and palatability [56, 57, 91, 92]. Here we show bilateral CeA CART neutralisation specifically reduced alcohol, but not sweetened alcohol bingeing in female mice. Notably, manipulating the CART system is challenging as there is no known cognate receptor(s), thus we relied on the administration of CART antibody to neutralise signalling which can have its limitations. However, supporting the validity of this approach, the effect seen on alcohol binge drinking in female mice was highly specific, as when one or more cannula were placed adjacent to the CeA, no effect was observed, suggesting bilateral inhibition of CART signalling is required to reduce alcohol consumption. Further, we saw no effect in male mice with CART neuralisation within the CeA, suggesting this is specific to females. Together, our data suggest the CeA may in part, underpin the action of CART in alcohol binge drinking in female mice; however, given the relatively small magnitude of the reduction, other brain regions are also likely involved. Further, whether this effect is driven by CART produced and released within the CeA itself, or from CART projections into the CeA requires greater examination. The CeA is a highly heterogenous population, historically distinguished by their expression of PKCδ or somatostatin [30, 93]. CeA CART cells primarily located in the capsular and lateral divisions of the CeA make up a subpopulation of PKCδ expressing cells (~35%) [30, 93]. The capsular and lateral CeA form inhibitory microcircuits that regulate output via the medial CeA subdivision, however PKCδ/CART cells also project directly outside of the CeA [94]. CeA PKCδ + cells receive direct input from the insula cortex and aversion sensing calcitonin gene-related peptide (CGRP) neurons in the lateral parabrachial nucleus [95] and densely express the CGRP receptor (calcitonin receptor-like, CALCRL) [96] suggesting a plausible mechanism in which CeA CART signalling could contribute to the encoding of aversive signals. However, whether this is driven through the CART expressing subpopulation of PKCδ has not been explored. Future studies are required to understand the nuanced neurochemical mechanisms underpinning these genotype x sex differences.

## Conclusions

To summarise, we have identified a novel role for the neuropeptide CART driving binge drinking in a sexually dimorphic manner. Specifically, CART deletion drove up binge drinking in male mice, while conversely reducing binge drinking in females. Further, our data identify a novel mechanism through which CART mediates binge drinking in female mice. We show CART’s role in binge drinking is not dependent on peripherally circulating sex steroid hormones, nor is it influenced by changes in anxiety-like or depressive-like behaviours, but appears to be driven by changes in bitter taste sensitivity. Finally, we identify the CeA as a node within the brain that mediates CART’s actions in driving excessive alcohol consumption specifically in female mice. Future research targeting the CART neuropeptide system may provide novel insights for AUD treatments that may have heightened efficacy in women.

### Supplementary information

Supplementary material
