# Supplementary material for: Cocaine and amphetamine regulated transcript (CART) mediates sex differences in binge drinking through central taste circuits
Source: Neuropsychopharmacology. 2023 Aug 22;49(3):541–50. doi: 10.1038/s41386-023-01712-2 (PMC10789734; doi:10.1038/s41386-023-01712-2)
Supplement: Supplementary file 1 — Supplementary material [file 41386_2023_1712_MOESM1_ESM.docx]

**Supplementary information**

**Cocaine- and amphetamine-regulated transcript (CART) mediates sex differences in binge drinking through central taste circuits**

Xavier J. Maddern, Bethany Letherby, Sarah S. Ch’ng, Amy Pearl, Andrea Gogos, Andrew J. Lawrence, & Leigh C. Walker

**Supplementary methods & materials**

***Animals***

Mice were single-housed and maintained in a temperature controlled room (21°C) under a 12hr light/dark cycle (light on or off at 7am) with *ad libitum* access to food (laboratory chow, Barastoc) and water. All mice were acclimated to the experimental holding room for one week prior to the commencement of experimentation, and were ~8 weeks of age at the beginning of experimentation.

***Genotyping***

CART KO and WT mice were verified through PCR (Figure S1A). Genomic DNA from WT and CART KO mice was isolated from tail samples and subjected to PCR procedures as previously described [1]. Briefly, PCR was performed using the primer sets: CART-A (5′-GCTGCCTACA GACGGCTGAC-3′) with Cre-D (5′-ACATCTTCAGGTTCTGCG-3′), CART-A with CART-D (5′-GGAGCTCTCCATGGTTCTGG-3). PCR with CART-A/Cre-D was designed to produce 430 bp fragments representing deletion of the entire CART gene to be expected in CART KO samples. The genotypes were further examined with PCR primed by CART-A/CART-D oligos for the generation of 172 bp amplicons representing part of the CART gene sequence to be expected in WT samples. Therefore, the combination of these two PCR reactions revealed homozygous CART KO, heterozygous (CART – not used in this study) and CART WT mice.

***Immunohistochemistry***

CART protein levels were also validated by immunohistochemistry (Figure S1B). Mice were deeply anaesthetised with pentobarbitone (80 mg/kg; i.p.) and transcardially perfused with 12mL of phosphate buffered saline (PBS) (0.1M, pH 7.4) followed by 50mL of 4% w/v PFA in PBS. Mice were then decapitated, brains removed and post-fixed in 4% w/v PFA solution for 2hr at 4°C. Brains were then transferred to 20% w/v sucrose in PBS for ~24hr at 4°C. Coronal sections (40 μm) were cut in a 1 in 4 series on a cryostat at -18°C (Cryocut 1800, Leica Microsystems, Heerbrugg, Switzerland) and stored in PBS with 0.1% sodium azide (Sigma-Aldrich).

Fluorescent Immunohistochemistry was used to validate CART KO (n = 2 male and 2 female/genotype). Sections were pre-blocked in 10% normal donkey serum (NDS) and 0.5% TX-100 in PBS at room temperature (RT) for 1hr. Sections were then incubated in a primary antibody solution containing rabbit anti-CART (1:1000; H-003-H2; Phoenix pharmaceuticals; Burlingame, CA, USA), 2% NDS, 0.1% TX-100 in PBS. Following incubation sections were washed 3 x 5min in PBS and incubated at RT for 2 hr in a secondary antibody solution of donkey anti-rabbit Alexa-fluor 488 (A150077, Life Technologies, Carlsbad, CA, USA), 2% NDS, 0.1% TX-100 in PBS. Finally, sections were washed 3 x 5min, mounted on microscope slides and coverslipped with fluorescence mounting medium (DAKO, Carpentaria, CA, USA).

To digitally capture CART fluorescence, a LSM 780 Zeiss Axio Imager 2 confocal laser scanning microscope (Carl Zeiss AG, Jena, Germany) using the same settings with a 20x objective for all mice (CART KO and WT), was used to obtain images of the lateral hypothalamus (LH), CeA and arcuate nucleus (Arc), which were quantified from three sections per mouse (Bregma -1.22 to -1.58 mm) [2] using image J (National Institutes of Health; RRID:SCR_003070).

***g/kg consumed calculation***

Total consumption in grams was calculated using the volume consumed in millilitres (mL), multiplied by the density of 10% v/v ethanol or 5% w/v sucrose. The grams per kilogram (g/kg) was calculated as follows; total grams consumed/(weight of mouse/1000).

***Light-dark box test***

Following continuous access ethanol two-bottle choice, a 10min light-dark (L/D) box test was employed to examine the role of CART and potential interactions of CART and alcohol in anxiety-like behaviour. Experiments were conducted on day 10 of alcohol abstinence between 10 am and 12 pm. Mice were placed in the 27.3cm (length) x 27.3cm (width) x 20.3cm (height) automated locomotor arena (Med Associates, St Albans, VT, USA). Half of the locomotor cell was covered by the ‘dark’ box made of plastic, opaque to visible light, but transparent to photo beams. A small opening (4 x 4cm) enabled mice to leave and enter the ‘dark’ side. The light side was lit by an array of light emitting diodes (~750 LUX in the centre), creating an aversive stimulus. Movements of the mice were tracked using activity monitor software (Med Associates). Test sessions commenced as the mice were placed into the dark half of the arena. The percentage time spent in the light side, latency to enter light (seconds) and number of entries into light were recorded.

***Saccharin Preference Test (SPT)***

To examine the role of CART and potential interactions of CART and alcohol on anhedonia-like behaviour during a single 12hr overnight period on day 14 of alcohol abstinence, mice had access to two-bottles containing 0.1% (w/v) saccharin solution or water [3]. The total volume of fluid intake was recorded, and preference ratio was calculated by calculating the volume of saccharin solution consumed as a proportion of total fluid intake.

***Porsolt swim test (PST)***

To examine the role of CART and potential interactions of CART and alcohol in depressive-like behaviours a PST was conducted on day 18 of alcohol abstinence, mice were individually placed into beakers (17 cm diameter) of water (23–25 °C) filled to a depth such that tails would not be in contact with the bottom of the beaker. Each test lasted for a total of 5min and was video recorded for subsequent scoring via depression scale program (CleaverSys, Reston, VA, USA). The total immobility time and latency to floating adopted by each mouse were recorded and percentage of time spent immobile was calculated.

***Ovariectomy surgery procedure***

Mice were anesthetized with isoflurane (5% induction; 2% maintenance). The surgical site was swabbed with chlorhexidine solution and shaved, and a small incision was made along the midline of the lower back, with the skin and underlying muscle being separated. The ovarian fat pad was located and an incision was made superiorly through the muscle wall. The ovary was then isolated and extracted through this incision, and excised. A single horizontal mattress suture closed the small incision in the muscle wall. The SHAM surgery was identical to that of the ovariectomy surgery, except the ovary was not disturbed nor removed. Following suturing, betadine antiseptic was applied over the sutures, and mice were administered with meloxicam (3mg/kg^-1^, s.c.) for analgesia, the antibiotic baytril (3mg/kg^-1^, s.c.), and saline (10ml/kg^-1^, s.c.) to facilitate recovery. Mice were given at least 7 days to recover, with body weights and health monitored before recommencing binge drinking training.

***Stereotaxic implantation of cannulae into the CeA***

Mice were anesthetized with isoflurane (5% induction; 2% maintenance), and placed in a stereotaxic frame (Stoelting Instruments). The surgical site was shaved and cleaned with betadine, and a small incision was made along the midline to expose the skull. Two holes were drilled into the skull through which screws (PlasticsOne) were inserted to anchor the cannula to the skull. Single-guide cannulas (26G, cut 5mm below pedestal; PlasticsOne) were implanted 1.2mm above the CeA (anteroposterior, -1.45mm; mediolateral, ±2.75; dorsoventral, -3.75). Dental cement (GAENIAL Universal Flo, Harry Schein, Mascot, NSW, Australia) was used to fix the cannula to the skull, and their patency was maintained through the insertion of a dummy cannula (PlasticsOne). Mice were administered with meloxicam (3mg/kg^-1^, s.c.) for analgesia, the antibiotic baytril (3mg/kg^-1^, s.c.), and saline (10ml/kg^-1^, s.c.) to facilitate recovery. Mice were given at least 7 days to recover from surgery prior to recommencement of binge drinking retraining.

***Intracranial infusions***

CART antibody or vehicle (0.5µl/hemisphere) was infused bilaterally at 0.25µl/min using injectors projecting 1.2mm below the guide cannula tips connected to polyethylene tubing attached to two 1µl microsyringes (SGE Analytical Science), connected to an automated syringe pump (Harvard Apparatus). Following the 2min infusion, the injectors remained in place for an additional 2min to minimize infusion spread and backtracking. Mice were then returned to their home-cage and the 10% v/v ethanol bottle was provided to commence the test session.

Following behavioural experimentation, mice were anesthetized (pentobarbitone 100mg/kg^-1^, i.p., Virbac) and methylene blue (0.5µL/ hemisphere) was infused through the cannula to validate surgical placement. Brains were extracted and frozen over dry ice, then were cut into 40µm coronal sections (Leica Microsystems cryostat) and counterstained with Neutral Red solution (Sigma-Aldrich). Cannula placements were verified by an investigator blinded to the behavioural data. A “hit” was defined as successful bilateral implantation of the cannulae into the CeA, whilst an anatomic control was defined as at least one cannula being implanted outside to the CeA.

***Statistical analysis***

For Experiment 1, the effect of genotype and session on ethanol, sucrose and ethanol + sucrose intake during binge drinking training were assessed using a repeated measures (RM) two-way analysis of variance (ANOVA). A two-tailed unpaired t-test assessed the effect of genotype on total g/kg consumption during the binge drinking test, and a RM two-way ANOVA examined the effect of genotype and time on the cumulative g/kg intake. Additionally, the average intake in g/kg of male and female WT littermates for each hour of the test session was calculated, with this being used to determine the delta intake of CART KO mice. A RM two-way ANOVA assessed the effect of sex and time on delta intake (g/kg) in CART KO mice. For Experiment 2, RM two-way ANOVAs assessed the effect of genotype and ethanol or sucrose concentration on consumption (g/kg), preference and total fluid intake (mL). For Experiment 3, RM two-way ANOVAs assessed the effect of genotype and tastant concentration on consumption (g/kg or mL), preference and total fluid intake (mL). For Experiment 4, a RM two-way ANOVA examined the effect of surgery group and session on ethanol consumption in g/kg pre- and post-surgery during binge drinking training. A two-tailed unpaired t-test assessed the effect of surgery group on total ethanol g/kg intake during the binge drinking test, and a RM two-way ANOVA examined the effect of time and surgery group on cumulative ethanol g/kg intake. A two-tailed unpaired t-test assessed the effect of surgery group on uterine tube weights. For Experiment 5, two-tailed paired t-tests assessed the effect of bilateral CART Ab infusion on ethanol, and sucrose supplemented ethanol, binge drinking. For experiment all analyses were performed using GraphPad Prism. Data are presented as mean ± standard error mean (SEM) and significance set at *p* < 0.05.

**Supplementary Figures:**

***Supplementary figure 1.*** *CART mouse validation. (A) PCR genotyping results for CART WT, HET and KO animals indicated by expression of the WT band at 172bp or mutant band at 430bp. Global CART KO does not alter body weight in (B) male or (C) female mice. RM Two-way ANOVA revealed an effect of time on weight in both sexes (Males, F_(9,306)_ = 14.93; p<0.0001) (Females, F_(9, 288)_ = 23.96; p<0.0001), but no effect of genotype on weight were observed for either sex (Males, F_(1,34)_ = 1.294; p = 0.263) (Females, F _(1,32)_ = 0.1131; p = 0.739) and no time x genotype interaction was seen (Males, F_(9, 306)_ = 0.7831, p = 0.635) (Females, (F_(9, 288)_ = 1.406, p = 0.185). Representative fluorescence micrographs of CART expression within the (D_1-2_) central nucleus of the amygdala (CeA), (E_1-2_) lateral hypothalamus (LH) and (F_1-2_) arcuate nucleus (Arc) in CART WT (n = 4) and KO (n = 4) mice. Students t-test showed a significant reduction in CART expression in all regions (CeA t_(6)_ =5.054, p = 0.0023; LH t_(6)_ =16.44, p <0.0001; Arc t_(6)_ =6.973, p = 0.0004).*


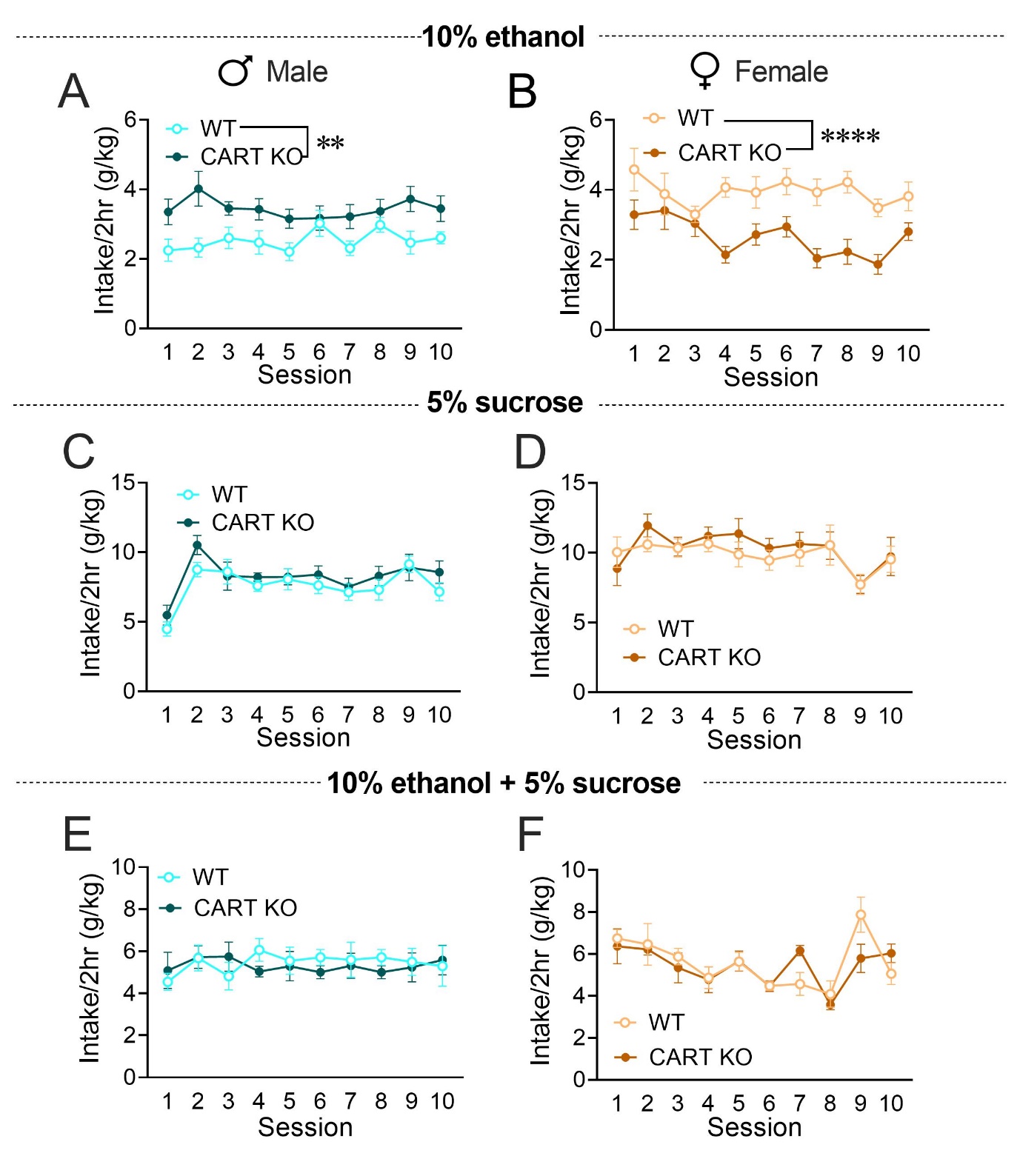


***Supplementary figure 2.*** *Binge drinking training for Experiment 1.* (A) RM two-way ANOVA revealed an effect of genotype with CART KO males having increased ethanol intake compared to WT littermates (*F*_(1,16)_ = 8.772, *p* = 0.009) and a session x genotype interaction (*F*_(9,144)_ = 1.321, *p* = 0.231), whilst there was no effect of session (*F*_(9,144)_ = 0.900, *p* = 0.527). (B) RM two-way ANOVA found an effect of genotype (*F*_(1,16)_ = 35.48, *p* < 0.0001) with CART KO females having reduced ethanol intake compared to WT littermates and an effect of session (*F*_(9,144)_ = 1.947, *p* = 0.0497), whilst there was no genotype x session interaction (*F*_(9,144)_ = 1.335, *p* = 0.224). (C) RM two-way ANOVA revealed no difference in sucrose intake between male CART KO and WT mice during training (*F*_(1,14)_ = 1.084, *p* = 0.315) and no genotype x session interaction (*F*_(9,126)_ = 0.771, *p* = 0.644), whilst there was an effect of session (*F*_(9,126)_ = 10.19, *p* < 0.001). (D) RM two-way ANOVA also showed no difference in female CART KO and WT mice sucrose intake during training (*F*_(1,15)_ = 0.2023, *p* = 0.6593) and no genotype x session interaction (*F*_(9,135)_ = 0.6598, *p* = 0.7439), whilst there was an effect of session (*F*_(9,135)_ = 4.350, *p* < 0.0001). (E) RM two-way ANOVA found no difference in male CART KO and WT mice 10% v/v ethanol + 5% w/v sucrose intake (*F*_(1,8)_ = 0.217, *p* = 0.654), and no genotype x session interaction (*F*_(9,72)_ = 0.503, *p* = 0.868) or effect of session (*F*_(2.558, 20.47)_ = 0.285, *p* = 0.805). (F) RM two-way ANOVA revealed no difference in female CART KO and WT mice 10% v/v ethanol + 5% w/v sucrose intake (*F*_(1,9)_ = 0.0741, *p* = 0.7916), and no genotype x session interaction (*F*_(9,81)_ = 1.955, *p* = 0.0555), whilst there was a main effect of session (*F*_(9,81)_ = 7.367, *p* < 0.0001).


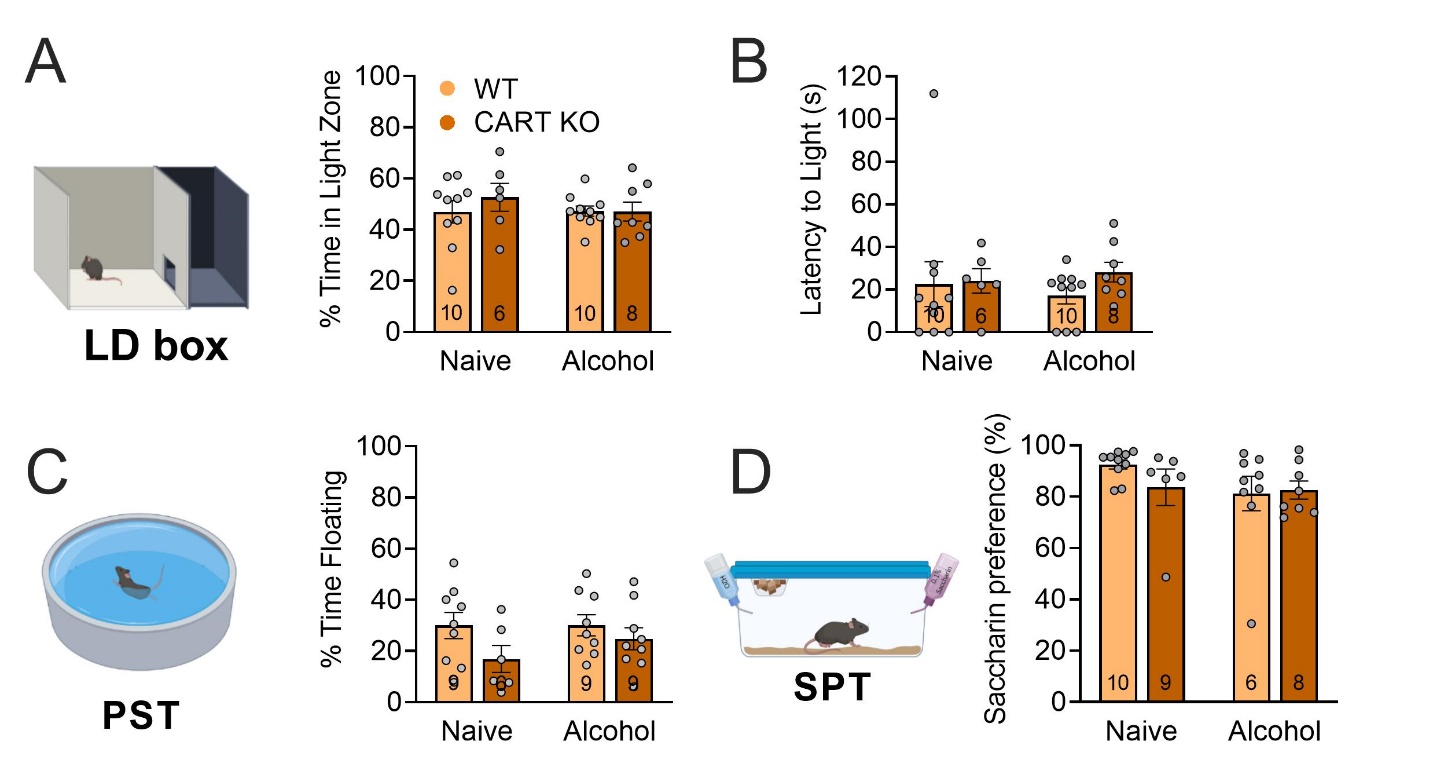


***Supplementary figure 3.*** *Assessment of other potential factors influencing binge drinking in female CART KO mice.* (A) A two-way ANOVA revealed no effect of alcohol experience (*F*_(1,30)_ = 0.453, *p* = 0.506), genotype (*F*_(1,30)_ = 0.486, *p* = 0.491), or alcohol experience x genotype interaction (*F*_(1,30)_ = 0.571, *p* = 0.456) on the percentage of time spent in the light zone during the light-dark box test. (B) A two-way ANOVA also found no effect of alcohol experience (*F*_(1,30)_ = 0.007, *p* = 0.932), genotype (*F*_(1,30)_ = 0.705, *p* = 0.408), or alcohol experience x genotype interaction (*F*_(1,30)_ = 0.406, *p* = 0.529) on the latency to enter the light zone during the light dark-box test. (C) A two-way ANOVA found no effect of alcohol experience (*F*_(1,29)_ = 0.698, *p* = 0.410), genotype (*F*_(1,29)_ = 3.708, *p* = 0.064), or alcohol experience x genotype interaction (*F*_(1,29)_ = 0.678, *p* = 0.417) on the percentage of time spent floating in the Porsolt swim test. (D) A two-way ANOVA showed no effect of alcohol experience (*F*_(1,29)_ = 1.555, *p* = 0.222), genotype (*F*_(1,29)_ = 0.568, *p* = 0.457), or alcohol experience x genotype interaction (*F*_(1,29)_ = 1.050, *p* = 0.314) on saccharin preference.


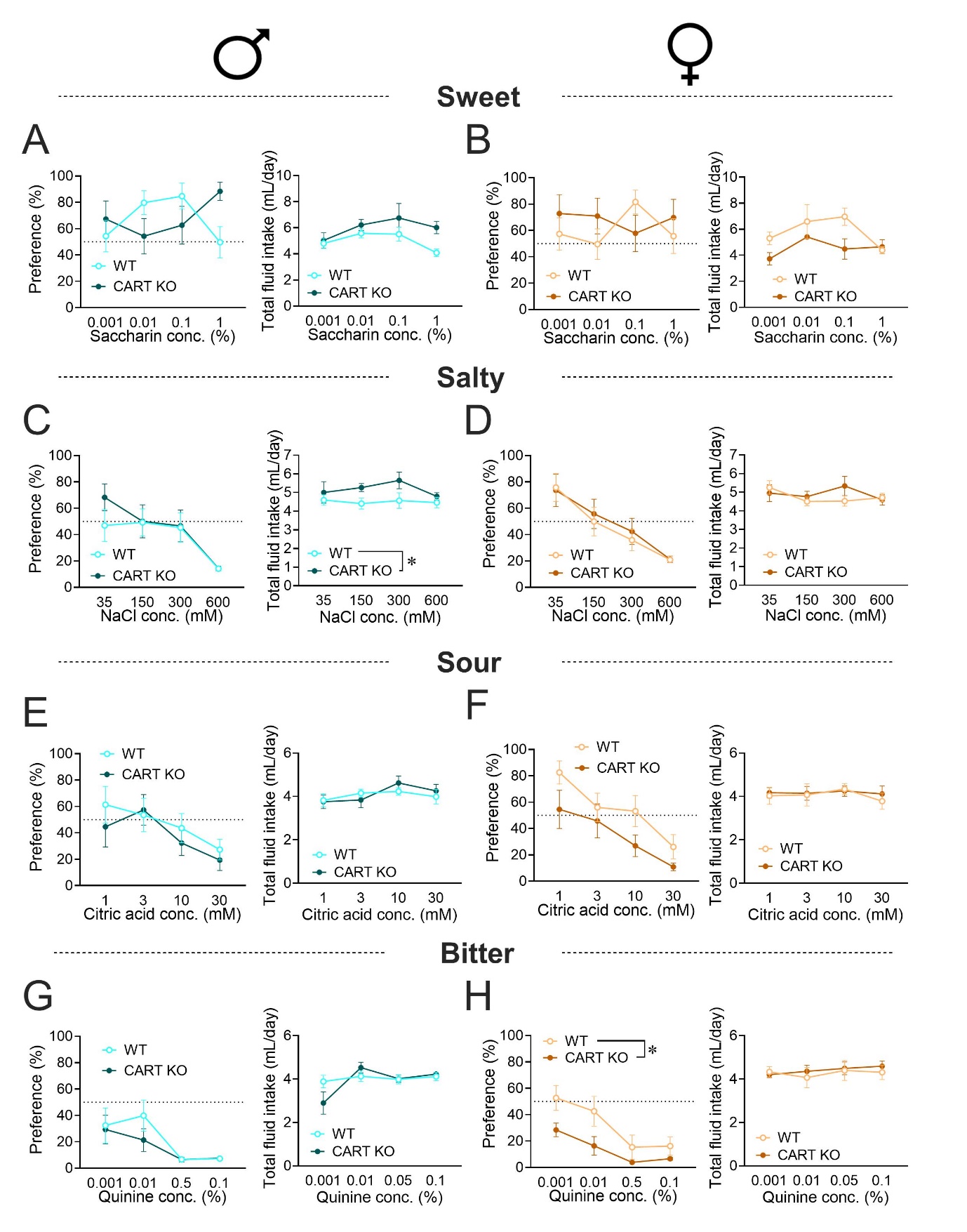


***Supplementary figure 4.*** *Preference and total fluid intake for the solutions presented to male and female CART KO and WT mice in Experiment 3.* (A) RM two-way ANOVA revealed a concentration x genotype interaction (*F*_(3,48)_ = 3.881, *p* = 0.015), whilst there was no effect of concentration (*F*_(2.447,39.16)_ = 0.475, *p* = 0.663) and genotype (*F*_(1,16)_ = 0.012, *p* = 0.916) on saccharin preference in male mice (left). RM two-way ANOVA found no effect of concentration (*F*_(1.954,31.27)_ = 2.817, *p* = 0.076), genotype (*F*_(1,16)_ = 4.021, *p* = 0.062), nor concentration x genotype interaction (*F*_(3,48)_ = 1.052, *p* = 0.378) on total fluid intake in male mice when presented with saccharin (right). (B) RM two-way ANOVA showed no effect of concentration (*F*_(2.868,45.89)_ = 0.219, *p* = 0.875), genotype (*F*_(1,16)_ = 0.454, *p* = 0.510), nor concentration x genotype interaction (*F*_(3,48)_ = 1.438, *p* = 0.243) on saccharin preference in female mice (left). RM two-way ANOVA revealed an effect of saccharin concentration on total fluid intake in female mice (*F*_(2.384,38.14)_ = 3.250, *p* = 0.042), whilst there was no effect of genotype (*F*_(1,16)_ = 3.545, *p* = 0.078) nor a concentration x genotype interaction (*F*_(3,48)_ = 1.724, *p* = 0.175) on total fluid intake (right). (C) RM two-way ANOVA showed an effect of concentration on salt preference in male mice (*F*_(2.658,42.53)_ = 8.785, *p* = 0.0002) with preference decreasing across increasing concentrations, whilst no effect of genotype (*F*_(1,16)_ = 0.480, *p* = 0.499) nor a concentration x genotype interaction (*F*_(3,48)_ = 0.645, *p* = 0.590) was seen (left). RM two-way ANOVA revealed an effect of genotype on total fluid intake in male mice when presented with salt (*F*_(1,16)_ = 4.843, *p* = 0.043), with male CART KO mice having increased total fluid intake, whilst no effect of concentration (*F*_(2.246,35.94)_ = 0.728, *p* = 0.505) nor a concentration x genotype interaction (*F*_(3,48)_ = 0.588 *p* = 0.653) were found (right). (D) RM two-way ANOVA showed an effect of NaCl concentration on preference (*F*_(3,48)_ = 12.36, *p* < 0.0001) with preference toward NaCl decreasing across increasing concentrations of NaCl in female mice, whilst no effect of genotype (*F*_(1,16)_ = 0.159, *p* = 0.695) nor a concentration x genotype interaction (*F*_(3,48)_ = 0.108, *p* = 0.955) were seen (left). RM two-way ANOVA revealed no effect of concentration (*F*_(2.597,41.56)_ = 1.258, *p* = 0.300), genotype (*F*_(1,16)_ = 0.256, *p* = 0.617) nor a concentration x genotype interaction (*F*_(3,48)_ = 1.484, *p* = 0.231) on total fluid intake in female mice when presented with NaCl (right). (E) RM two-way ANOVA showed preference toward citric acid to decrease in male mice across increasing concentrations of citric acid (*F*_(2.585,41.37)_ = 4.944, *p* = 0.007), whilst no effect of genotype (*F*_(1,16)_ = 0.491, *p* = 0.494) nor a concentration x genotype interaction (*F*_(3,48)_ = 0.422, *p* = 0.738) were seen (left). RM two-way ANOVA revealed no effect of concentration (*F*_(2.438,39.02)_ = 2.172, *p* = 0.118), genotype (*F*_(1,16)_ = 0.072, *p* = 0.793), nor a concentration x genotype interaction (*F*_(3,48)_ = 0.792, *p* = 0.505) on total fluid intake in male mice when presented with citric acid (right). (F) RM two-way ANOVA found a reduced preference toward citric acid in female mice across increasing concenrations (*F*_(2.024,32.39)_ = 13.45, *p* < 0.0001), whilst no effect of genotype (*F*_(1,16)_ = 3.236, *p* = 0.091) nor a concentration x genotype interaction (*F*_(3,48)_ = 0.566, *p* = 0.640) were observed (left). RM two-way ANOVA showed no effect of concentration (*F*_(2.233,35.72)_ = 0.414, *p* = 0.686), genotype (*F*_(1,16)_ = 0.114, *p* = 0.740), nor a concentration x genotype interaction (*F*_(3,48)_ = 0.157, *p* = 0.925) on total fluid intake in female mice when presented with citric acid (right). (G) RM two-way ANOVA revealed male mice to have a reduced preference toward quinine across increasing concentrations (*F*_(1.739,27.82)_ = 7.475, *p* = 0.004), whilst no effect of genotype (*F*_(1,16)_ = 0.544, *p* = 0.471) nor a concentration x genotype interaction (*F*_(3,48)_ = 0.791, *p* = 0.505) were seen (left). RM two-way ANOVA showed an increase in total fluid intake in male mice across increasing concentrations of quinine (*F*_(1.638,26.21)_ = 4.872, *p* = 0.021), whilst no effect of genotype (*F*_(1,16)_ = 0.293, *p* = 0.596) nor a concentration x genotype interaction (*F*_(3,48)_ = 2.655, *p* = 0.059) were found (right). (H) RM two-way ANOVA revealed reduced preference for quinine in female CART KO mice compared to WT mice (*F*_(1,16)_ = 5.116, *p* = 0.038) and a reduced preference for quinine in female mice across increasing concentrations (*F*_(2.532,40.51)_ = 10.52, *p* < 0.0001), whilst no concentration x genotype interaction (*F*_(3,48)_ = 0.868, *p* = 0.464) was observed (left). RM two-way ANOVA showed no effect of concentration (*F*_(2.344,37.50)_ = 0.450, *p* = 0.672), genotype (*F*_(1,16)_ = 0.133, *p* = 0.720), nor a concentration x genotype interaction (*F*_(3,48)_ = 0.294, *p* = 0.830) on total fluid intake in female mice when presented with quinine (right).

***Supplementary table 1. Complete statistical analyses for Experiment 1***

|  | Statistical analysis | Results |
| --- | --- | --- |
| Male ethanol binge drinking test: Cumulative intake | RM two-way ANOVA | Genotype X Time interaction: *F*_(3,48)_ = 4.545, *p* = 0.007  Main effect of genotype: *F*_(1,16)_ = 13.96, *p* = 0.0018  Main effect of time: *F*_(3,48)_ = 188.1, *p* < 0.0001 |
| Male ethanol binge drinking test: Total intake | Two-tailed unpaired t-test | *t*_(16)_ = 3.346, *p* = 0.0041 |
| Female ethanol binge drinking test: Cumulative intake | RM two-way ANOVA | Genotype X Time interaction: *F*_(3,48)_ = 10.53, *p* < 0.0001 Main effect of genotype: *F*_(1,16)_ = 25.92, *p* = 0.0001 Main effect of time: *F*_(3,48)_ = 242.3, *p* < 0.0001 |
| Female ethanol binge drinking test: Total intake | Two-tailed unpaired t-test | *t*_(16)_ = 5.657, *p* < 0.0001 |
| Delta intake of ethanol during binge drinking test | RM two-way ANOVA | Sex X Time interaction: *F*_(3,54)_ = 22.91, *p* < 0.0001 Main effect of sex: *F*_(1,18)_ = 64.87, *p* < 0.0001 Main effect of time: *F*_(3, 54)_ = 2.328, *p* = 0.085 |
| Male sucrose binge drinking test: Cumulative intake | RM two-way ANOVA | Genotype X Time interaction: *F*_(3,42)_ = 0.720, *p* = 0.546 Main effect of genotype: *F*_(1,14)_ = 1.376, *p* = 0.260 Main effect of time: *F*_(3,42)_ = 261.4, *p* < 0.0001 |
| Male sucrose binge drinking test: Total intake | Two-tailed unpaired t-test | *t*_(14)_ = 1.031, *p* = 0.3201 |
| Female sucrose binge drinking test: Cumulative intake | RM two-way ANOVA | Genotype X Time interaction: *F*_(3,45)_ = 1.118, *p* = 0.3518 Main effect of genotype: *F*_(1,15)_ = 1.039, *p* = 0.3243 Main effect of time: *F*_(3,45)_ = 178.0, *p* < 0.0001 |
| Female sucrose binge drinking test: Total intake | Two-tailed unpaired t-test | *t*_(15)_ = 0.8134, *p* = 0.4287 |
| Delta intake of sucrose during binge drinking test | RM two-way ANOVA | Sex X Time interaction: *F*_(3,42)_ = 0.215, *p* = 0.886 Main effect of sex: *F*_(1,14)_ = 0.094, *p* = 0.764  Main effect of time: *F*_(3,42)_ = 2.863, *p* = 0.048 |
| Male ethanol + sucrose binge drinking test: Cumulative intake | RM two-way ANOVA | Genotype X Time interaction: *F*_(3,24)_ = 0.680, *p* = 0.573 Main effect of genotype: *F*_(1,8)_ = 0.230, *p* = 0.644 Main effect of time: *F*_(1.765,14.12)_ = 138.4, *p* < 0.0001 |
| Male ethanol + sucrose binge drinking test: Total intake | Two-tailed unpaired t-test | *t*_(8)_ = 0.0475, *p* = 0.9633 |
| Female ethanol + sucrose binge drinking test: Cumulative intake | RM two-way ANOVA | Genotype X Time interaction: *F*_(3,27)_ = 1.447, *p* = 0.2511 Main effect of genotype: *F*_(1,9)_ = 0.00027, *p* = 0.9872 Main effect of time: *F*_(3, 27)_ = 255.8, *p* < 0.0001 |
| Female ethanol + sucrose binge drinking test: Total intake | Two-tailed unpaired t-test | *t*_(9)_ = 0.3298, *p* = 0.7491 |
| Delta intake of alcohol + sucrose during binge drinking test | RM two-way ANOVA | Sex X Time interaction: *F*_(3,24)_ = 2.745, *p* = 0.065 Main effect of sex: *F*_(1,8)_ = 0.063, *p* = 0.809  Main effect of time: *F*_(3,24)_ = 1.469, *p* = 0.248 |

***Supplementary table 2. Complete statistical analyses for Experiment 2.***

|  | Statistical analysis | Results |
| --- | --- | --- |
| Male ethanol two-bottle choice intake | RM two-way ANOVA | Genotype X Ethanol concentration interaction: *F*_(3,48)_= 1.253, *p* = 0.3012 Main effect of genotype: *F*_(1,16)_ = 2.313, *p* = 0.148 Main effect of ethanol concentration: *F*_(2.043,32.69)_ = 51.69, *p* < 0.0001 |
| Male ethanol two-bottle choice preference | RM two-way ANOVA | Genotype X Ethanol concentration interaction: *F*_(3,51)_ = 1.496, *p* = 0.2269 Main effect of genotype: *F*_(1,17)_ = 7.814, *p* = 0.0124 Main effect of ethanol concentration: *F*_(2.434,41.38)_ = 18.93, *p* < 0.0001 |
| Male two-bottle choice total fluid intake | RM two-way ANOVA | Genotype X Ethanol concentration interaction: *F*_(3,48)_ = 0.172, *p* = 0.915 Main effect of genotype: *F*_(1,16)_ = 31.72, *p* < 0.0001 Main effect of ethanol concentration: *F*_(1.245,19.91)_ = 15.03, *p* = 0.0005 |
| Female ethanol two-bottle choice intake | RM two-way ANOVA | Genotype X Ethanol concentration interaction: *F*_(3,48)_= 3.277, *p* = 0.0288 Main effect of genotype: *F*_(1,16)_ = 14.67, *p* = 0.0015 Main effect of ethanol concentration: *F*_(3,48)_ = 42.45, *p* < 0.0001 |
| Female ethanol two-bottle choice preference | RM two-way ANOVA | Genotype X Ethanol concentration interaction: *F*_(3,48)_ = 8.924, *p* < 0.0001 Main effect of genotype: *F*_(1,16)_ = 23.08, *p* = 0.0002 Main effect of ethanol concentration: *F*_(1.639, 26.22)_ = 11.85, *p* = 0.0004 |
| Female two-bottle choice total fluid intake | RM two-way ANOVA | Genotype X Ethanol concentration interaction: *F*_(3,48)_ = 0.304, *p* = 0.822 Main effect of genotype: *F*_(1,16)_ = 0.003, *p* = 0.9593 Main effect of ethanol concentration: *F*_(1.838,29.4)_ = 0.501, *p* = 0.596 |
| Female ethanol + sucrose two-bottle choice intake | RM two-way ANOVA | Genotype X Ethanol concentration interaction: *F*_(2,32)_ = 1.786, *p* = 0.1839 Main effect of genotype: *F*_(1,16)_ = 4.475, *p* = 0.0447 Main effect of sucrose concentration: *F*_(1.905,30.48)_ = 19.39, *p* < 0.0001 |
| Female ethanol + sucrose two-bottle choice preference | RM two-way ANOVA | Genotype X Ethanol concentration interaction: *F*_(2,32)_ = 6.919, *p* = 0.0032 Main effect of genotype: *F*_(1,16)_ = 4.803, *p* = 0.0435 Main effect of sucrose concentration: *F*_(1.622,25.95)_ = 24.82, *p* < 0.0001 |
| Female ethanol + sucrose total fluid intake | RM two-way ANOVA | Genotype X Ethanol concentration interaction: *F*_(2,32)_ = 0.119, *p* = 0.888 Main effect of genotype: *F*_(1,16)_ = 1.124, *p* = 0.3047 Main effect of ethanol concentration: *F*_(1.81,28.97)_ = 9.484, *p* = 0.0009 |

***Supplementary table 3. Complete statistical analyses for Experiment 3.***

|  | Statistical analysis | Results |
| --- | --- | --- |
| Male saccharin two-bottle choice intake | RM two-way ANOVA | Genotype X Saccharin concentration interaction: *F*_(3,48)_ = 4.008, *p* = 0.013 Main effect of genotype: *F*_(1,16)_ = 0.360, *p* = 0.557 Main effect of saccharin concentration: *F*_(1.716,27.45)_ = 1.020, *p* = 0.363 |
| Female saccharin two-bottle choice intake | RM two-way ANOVA | Genotype X Saccharin concentration interaction: *F*_(3,48)_ = 0.359, *p* = 0.782 Main effect of genotype: *F*_(1,16)_ = 0.195, *p* = 0.665 Main effect of saccharin concentration: *F*_(1.828,29.25)_ = 0.710, *p* = 0.488 |
| Male NaCl two-bottle choice intake | RM two-way ANOVA | Genotype X NaCl concentration interaction: *F*_(3,48)_ = 0.557, *p* = 0.646 Main effect of genotype: *F*_(1,16)_ = 0.070, *p* = 0.795 Main effect of NaCl concentration: *F*_(2.349,37.59)_ = 8.098, *p* = 0.0007 |
| Female NaCl two-bottle choice intake | RM two-way ANOVA | Genotype X NaCl concentration interaction: *F*_(3,48)_ = 0.275, *p* = 0.843 Main effect of genotype: *F*_(1,16)_ = 0.227, *p* = 0.640 Main effect of NaCl concentration: *F*_(2.005,3208)_ = 10.02, *p* = 0.0004 |
| Male citric acid two-bottle choice intake | RM two-way ANOVA | Genotype X Citric acid concentration interaction: *F*_(3,48)_ = 0.161, *p* = 0.922 Main effect of genotype: *F*_(1,16)_ = 0.599, *p* = 0.450 Main effect of citric acid concentration: *F*_(2.498,39.97)_ = 3.410, *p* = 0.034 |
| Female citric acid two-bottle choice intake | RM two-way ANOVA | Genotype X Citric acid concentration interaction: *F*_(3,48)_ = 1.170, *p* = 0.331 Main effect of genotype: *F*_(1,16)_ = 1.807, *p* = 0.198 Main effect of citric acid concentration: *F*_(2.365,37.84)_ = 12.00, *p* < 0.0001 |
| Male quinine two-bottle choice intake | RM two-way ANOVA | Genotype X Quinine concentration interaction: *F*_(3,48)_ = 0.765, *p* = 0.519 Main effect of genotype: *F*_(1,16)_ = 1.243, *p* = 0.281 Main effect of quinine concentration: *F*_(1.687,26.99)_ = 6.090, *p* = 0.009 |
| Female quinine two-bottle choice intake | RM two-way ANOVA | Genotype X Quinine concentration interaction: *F*_(3,48)_ = 1.041, *p* = 0.383 Main effect of genotype: *F*_(1,16)_ = 6.954, *p* = 0.018 Main effect of quinine concentration: *F*_(2.03,32.47)_ = 10.41, *p* = 0.0003 |
| Female quinine + sucrose two bottle-choice intake | RM two-way ANOVA | Genotype X Sucrose concentration interaction: *F*_(2,32)_ = 2.237, *p* = 0.123 Main effect of genotype: *F*_(1,16)_ = 0.295, *p* = 0.595 Main effect of sucrose concentration: *F*_(1.149,18.39)_ = 25.32, *p* < 0.0001 |
| Female quinine + sucrose two-bottle choice preference | RM two-way ANOVA | Genotype X Sucrose concentration interaction: *F*_(2,32)_ = 6.510, *p* = 0.004 Main effect of genotype: *F*_(1,16)_ = 0.440, *p* = 0.517 Main effect of sucrose concentration: *F*_(1.408,22.52)_ = 23.73, *p* < 0.0001 |

***Supplementary table 4. Complete statistical analyses for Experiment 4.***

|  | Statistical analysis | Results |
| --- | --- | --- |
| Binge drinking training pre-surgery | RM two-way ANOVA | Surgery group X Session interaction: *F*_(9,160)_ = 0.338, *p* = 0.961  Main effect of treatment group: *F*_(1,18)_ = 0.752, *p* = 0.397  Main effect of session: *F*_(4.241,75.39)_ = 11.63, *p* < 0.0001 |
| Binge drinking training post-surgery | RM two-way ANOVA | Surgery group X Session interaction: *F*_(3,47)_ = 0.272, *p* = 0.845  Main effect of treatment group: *F*_(1,18)_ = 0.936, *p* = 0.346  Main effect of session: *F*_(2.14,33.59)_ = 4.405, *p* = 0.018 |
| Binge drinking test: Cumulative intake | RM two-way ANOVA | Surgery group X Time interaction: *F*_(3,54)_ = 0.7834, *p* = 0.5084  Main effect of treatment group: *F*_(1,18)_ = 0.2239, *p* = 0.6417  Main effect of time: *F*_(1.864,33.55)_ = 508.2, *p* < 0.0001 |
| Binge drinking test:  Total intake | Two-tailed unpaired t-test | *t*_(18)_ = 0.5102, *p* = 0.6161 |
| Uterine tube weights | Two-tailed unpaired t-test | *t*_(18)_ = 14.27, *p* < 0.0001 |

***Supplementary table 5. Complete statistical analyses for Experiment 5.***

|  | Statistical analysis | Results |
| --- | --- | --- |
| Male ethanol binge drinking test: Total intake | Two-tailed paired t-test | *t*_(6)_ = 0.0163, *p* = 0.9875 |
| Male ethanol binge drinking test anatomical controls: Total intake | Two-tailed paired t-test | *t*_(6)_ = 0.9629, *p* = 0.3728 |
| Female ethanol binge drinking test: Total intake | Two-tailed paired t-test | *t*_(5)_ = 3.446, *p* = 0.0183 |
| Female ethanol binge drinking test anatomical controls: Total intake | Two-tailed paired t-test | *t*_(8)_ = 1.064, *p* = 0.3183 |
| Female sucrose + ethanol binge drinking test: Total intake | Two-tailed paired t-test | *t*_(9)_ = 0.4227, *p* = 0.6824 |
| Female sucrose + ethanol binge drinking test anatomical control: Total intake | Two-tailed paired t-test | *t*_(7)_ = 0.1258, *p* = 0.9034 |

**References**

1. Lau J, Shi Y-C, Herzog H. Temperature dependence of the control of energy homeostasis requires CART signaling. Neuropeptides. 2016;59:97-109.

2. Paxinos G, Franklin LB. The mouse brain in stereotaxic coordinates. 2nd ed. Academy Press; 2001.

3. Short JL, Ledent C, Drago J, Lawrence AJ. Receptor crosstalk: characterization of mice deficient in dopamine D1 and adenosine A2A receptors. Neuropsychopharmacology. 2006;31(3):525-34. doi:10.1038/sj.npp.1300852
